# Depressive Emotionality Moderates the Influence of the BDNF Val66Met Polymorphism on Executive Functions and on Unconscious Semantic Priming

**DOI:** 10.1007/s12031-020-01479-x

**Published:** 2020-01-30

**Authors:** Simon Sanwald, Christian Montag, Markus Kiefer

**Affiliations:** 1 Department of Psychiatry, Ulm University, Ulm, Germany; 2 Department of Molecular Psychology, Institute of Psychology and Education, Ulm University, Ulm, Germany

**Keywords:** Semantic priming, Genetics, Executive functions, Primary emotions, Depression

## Abstract

Automatic semantic processing can be assessed using semantic priming paradigms. Individual differences in semantic priming have been associated with differences in executive functions. The brain-derived neurotrophic factor (BDNF) Val66Met (substitution of valine (Val) to methionine (Met) at codon 66) polymorphism has been shown to be associated with executive functions as well as depression. Depression-associated variables such as depressed mood also moderated the relationship between BDNF Val66Met and executive functions in previous work. In this study, we therefore aimed at investigating whether BDNF Val66Met genotype modulates masked and unmasked semantic priming as well as executive functions and whether sadness is a moderator of these associations. We collected data of *N* = 155 participants measuring reaction times (RTs) as well as error rates (ERs) in masked and unmasked semantic priming paradigms using a lexical decision task. We assessed the primary emotion of SADNESS using the Affective Neuroscience Personality Scale (ANPS) and working memory using digit span forward and backward tasks. Met+ carriers showed reduced RT priming and increased ER priming in the masked priming paradigm. Even though there was no direct association between BDNF Val66Met and executive functions, SADNESS significantly moderated the association between BDNF Val66Met and executive functions as well as masked RT priming. We suggest that Met+ individuals with low depressive tendencies have not only superior EF, but also a faster and more superficial processing style, compared with Val/Val homozygotes. However, in Met+ individuals, cognitive functioning exhibits a greater vulnerability to depressed emotionality compared with Val/Val homozygotes. Our study thus demonstrates how emotional and molecular genetic factors exert an interacting influence on higher-level cognition.

**Electronic supplementary material:**

The online version of this article (10.1007/s12031-020-01479-x) contains supplementary material, which is available to authorized users.

## Introduction

Semantic word processing, i.e., accessing the meaning of words, can be investigated with the semantic priming paradigm (Meyer and Schvaneveldt 1971). In the semantic priming paradigm, a prime word presented prior to a semantically related target stimulus facilitates the response to the target (Neely 1977, 1991). For instance, responses in a lexical decision task (discrimination between words and pseudowords) are faster and more accurate, when prime and target words are semantically related. Semantic priming effects can reflect automatic as well as controlled semantic processing. Automatic semantic processing such as spreading activation (Collins and Loftus 1975) or preactivation of semantic features (Masson 1995) is thought to be assessed when using short stimulus onset asynchronies (SOAs) between prime and target (Neely 1991), whereas controlled mechanisms such as expectancy generation or semantic matching (a recognized semantic relation indicates a word response in the lexical decision task) are assumed to operate only at SOAs longer than 300 ms (Neely 1991). There are however indications that some controlled priming mechanisms such as semantic matching contribute also at short SOAs (Koivisto 1998). In order to investigate automatic semantic processing in isolation, the masked priming paradigm can be used (Kiefer 2002; Marcel 1983): A mask such as a random sequence of letters presented before and/or after the prime prevents its conscious perception (Breitmeyer and Öğmen 2006). As controlled priming mechanisms are assumed to depend on conscious prime identification (Neely 1991), priming elicited by unconsciously perceived masked stimuli must arise exclusively by automatic priming mechanisms.

The magnitude of both conscious and unconscious semantic priming differs considerably across individuals and has been previously related to interindividual differences in executive functions (EFs) in both patient and healthy population (Kiefer et al. 2005; Kiefer et al. 2009; Moritz et al. 1999; Moritz et al. 2003; Spitzer et al. 1993). EFs entail all top-down mental processes used to control or integrate other cognitive processes (Miyake et al. 2000). They are especially important in new or complex situations when cognitive routines do not yet exist or fail (Norman and Shallice 1986; Posner and Dehaene 1994). In support of the proposed association between executive functioning and semantic priming, thought-disordered patients with schizophrenia exhibited exaggerated unconscious priming for directly related words (e.g., hen-egg) compared with healthy control participants (Kiefer et al. 2009). These differences in priming have been explained by deficits in prefrontal neural circuits, which lead to impaired executive functioning in this psychiatric patient group (Kiefer et al. 2009). In healthy participants, individuals with low digit span backward performance as a measure of EF of working memory exhibited larger priming for visible indirectly (e.g., lemon-sweet) associated primes (Kiefer et al. 2005). Furthermore, semantic processing and EF have a partially overlapping neural substrate in prefrontal cortex, rendering an interaction between both cognitive functions likely (Norman and Shallice 1986; Ulrich et al. 2014; Ulrich et al. 2013; Wagner et al. 2001). Taken together, these findings suggest an overlapping neural substrate and a negative association between EF and semantic priming. Finally, even though classical theories of automaticity postulate automatic processes to occur independent of executive control mechanisms in a bottom-up–driven fashion (Posner and Snyder 1975), more recent theories (Kiefer and Martens 2010) and supporting evidence (Ansorge and Neumann 2005; Martens et al. 2011) indicate that even automatic semantic processing depends on cognitive control settings.

As interindividual differences in EF can in part be explained by genetically determined predispositions (Friedman et al. 2008), which influence brain structure and functioning, interindividual differences in semantic priming might depend on the same genetic influence. One earlier study (Reuter et al. 2009) investigated an association between semantic priming and the COMT Val158Met single nucleotide polymorphism (SNP), which alters the gene coding the enzyme catechol-O-methyltransferase (COMT). COMT catabolizes dopamine in prefrontal areas and has been shown to be related to different measures of EF (Friedman et al. 2008). However, in this earlier study (Reuter et al. 2009), COMT Val158Met only influenced the general response speed in the lexical decision task, but did not affect magnitude of visible unmasked and invisible masked semantic priming. Another potentially relevant SNP influencing EF is the brain-derived neurotrophic factor (BDNF) (Egan et al. 2003; Wang et al. 2012).

BDNF is the most widely distributed secretory neurotrophin in the mammalian brain (Barde et al. 1982) and is currently thought to regulate synaptic transmission and plasticity (Lu 2003). Further, BDNF is known to affect the growth and survival of neurons (Martinowich et al. 2007). On a molecular genetic level, there is a single nucleotide polymorphism which is located in the 5′pro domain of the BDNF gene on human chromosome 11p14.1. The exchange of guanine for adenine at position 196 results in an amino acid substitution of valine (Val) to methionine (Met) at codon 66 (Val66Met) in proBDNF (Hall et al. 2003). Like other neurotrophins, BDNF has two forms: proBDNF and matureBDNF. By proteolytical cleavage, proBDNF is converted into its mature form (Seidah et al. 1996). The two forms have their own preferred cognate receptors. While matureBDNF preferentially binds to tyrosin kinase B receptor (TrkB) in the hippocampus thereby triggering dendritic spine growth, proBDNF binding to p75^NTR^ receptors is associated with apoptosis (Lu et al. 2005). The homozygous 66Met variant of BDNF Val66Met is extremely rare occurring only in 2–3%, while the heterozygous Val/Met genotype occurs with a prevalence of 20 to 30% in the Caucasian population (Montag et al. 2010a; Shimizu et al. 2004). The polymorphism has been shown to alter intracellular trafficking and packaging of proBDNF and as a consequence the secretion of matureBDNF (Egan et al. 2003). The 66Met allele has been associated with reduced volume of the hippocampus or the medial temporal lobe in general (Bueller et al. 2006; Montag et al. 2009, 2010c) and sometimes but not consistently with depression (Verhagen et al. 2010). Integrity of the medial temporal lobe has been associated with semantic processing and depression (Gatt et al. 2009; McCarthy et al. 1995). Moreover, the projections connecting the hippocampus with areas of the prefrontal cortex have been shown to play an important role in cognition and memory (Laroche et al. 2000).

However, the findings considering the associations of BDNF Val66Met and cognitive functions are heterogeneous and concern a broad range of functions: On the one hand, the 66Met allele has been shown to be associated not only with a reduced volume of the medial temporal lobe, but also with a volume reduction of the prefrontal cortex (Montag et al. 2009; Pezawas et al. 2004), diminished hippocampal engagement during encoding and retrieval processes (Hariri et al. 2003), poorer episodic memory (Egan et al. 2003), impaired visual iconic memory (Beste et al. 2011) as well as impaired long-term memory (Montag et al. 2014). On the other hand, the 66Met allele has also been associated with enhanced task switching (Gajewski et al. 2011) and reduced stroop interference in elderly (Gajewski et al. 2012). However, a meta-analysis by Mandelman and Grigorenko (2012) did not show a significant association between BDNF Val66Met genotype and any of the examined behavioral phenotypes. More recently, several studies reported an interaction of BDNF Val66Met genotype and early life stress (ELS) in the prediction of hippocampal encoding activity (Molendijk et al. 2012), affective memory in males (van Oostrom et al. 2012), and working memory (Gatt et al. 2009). For example, there was a positive association between ELS and working memory performance for homozygous Val/Val carriers, whereas Met+ individuals showed poorer working memory performance as a function of ELS (Gatt et al. 2009). Moreover, depressive symptoms have been found to interact with BDNF Val66Met genotype predicting the time stability of information stored in iconic memory. The interaction was driven by a positive correlation of depressive symptoms and pre-attentive visual sensory memory performance in Val/Val homozygotes, whereas Met+ individuals did not show an association between depressive mood and memory performance (Beste et al. 2011). This interaction between the BDNF Val66Met genotype and depressive mood might explain the heterogeneity of the results concerning the association between BDNF Val66Met and EF, in which the moderating influence of mood was not considered.

Depressive personality traits can be assessed using the Affective Neuroscience Personality Scales (ANPS) (Davis et al. 2003; Montag and Davis 2018) built upon the conceptual framework of affective neuroscience comprising the fundamental emotional tendencies of all mammals: the primary emotions (Panksepp 2004). Primary emotions might represent the evolutionary oldest part of human personality (Montag and Panksepp 2017). Until now, seven primary emotions with intrinsic positive and negative valence have been identified using deep brain stimulation (Davis and Montag 2019; Delgado et al. 1954; Deris et al. 2017; MacLean and Delgado 1953; Olds and Milner 1954; Panksepp 1971, 2011). The SADNESS system, which is related to depression, is activated in situations of separation distress and substantiated by feelings of loneliness and sadness, eventually precipitating depression (Montag and Panksepp 2017). The neuroanatomical correlate of the primary emotion SADNESS is considered being hosted in the neural circuits of limbic areas (Deris et al. 2017; Montag and Panksepp 2017). A recent study showed a strong association between SADNESS scores and depression severity (Montag et al. 2017). In earlier research, the negative effect of depressed mood on cognitive abilities, however, has only been observed when examining EF (Snyder 2013) and not in tasks dependent on unconscious automatic processing (Hartlage et al. 1993).

In the present research, we therefore aimed at examining the associations between semantic priming, the BDNF Val66Met polymorphism, EF, and depressive personality traits. We collected data from 155 healthy participants assessing genotype with respect to the BDNF Val66Met polymorphism and primary emotions using the ANPS (Davis and Panksepp 2011). Semantic processing was probed with unmasked (Kiefer et al. 2005) and masked versions (Kiefer 2002) of the semantic priming paradigm within a lexical decision task, in order to capture semantic processing under aware and purely unconscious automatic conditions (similar to Reuter et al. 2009). Working memory performance was assessed using a digit span forward and backward task, the latter also reflecting executive functioning, for information has to be recalled in a manipulated way.

Consequently, since BDNF Val66Met genotype has been associated with alterations in a broad range of cognitive functions including EF (Gajewski et al. 2011; Montag et al. 2009, 2014) and since priming was negatively related to EF (Kiefer et al. 2005), we assumed the BDNF 66Met allele to be associated with larger priming effects and impaired working memory. Taking into account recent theories suggesting automatic semantic processing to depend on cognitive control settings (Kiefer and Martens 2010), we hypothesized the association between BDNF Val66Met and semantic priming to be mediated by working memory capacity. The BDNF Val66Met polymorphism on the other hand is presumed to interact with depressive personality traits regarding EF (Beste et al. 2011). Thus, we assumed SADNESS to moderate the association between BDNF Val66Met genotype and EF, i.e., performance in the digit span backward task. Last, we aimed to explore if SADNESS also moderates the association between BDNF Val66Met genotype and semantic priming.

## Methods

### Participants

For the present study, 188 participants were recruited from the database of the Ulm Gene Brain Behavior Project (UGBBP) on a voluntary basis. Inclusion criteria for the present study were German as mother tongue, normal or corrected-to-normal vision, and a completed ANPS. Exclusion criterion was a lifetime diagnosis of neurological or psychiatric disorders as well as other general health problems affecting functioning of the central nervous system according to self-report. Participants were initially screened for inclusion and exclusion criteria before entering the database of the UGBBP with a short online questionnaire. A more detailed additional screening was performed before the experimental session: Participants were interviewed by a trained psychologist with regard to psychiatric or neurological disorders as well as general health problems using a standardized in-house screening questionnaire. If a participant reported any kind of psychiatric or neurological disorder, or any other relevant health problem, the participant was excluded from further analysis. Based on this detailed interview, 15 participants were excluded from the initial sample because it turned out that they did not meet the inclusion and exclusion criteria: depression, 5 participants; mania, 1 participant; eating disorder, 1 participant; multiple sclerosis, 1 participant; traumatic head injury, 1 participant; and without response to the screening questions, 6 participants. Further, genotyping considering BDNF Val66Met had to be successful (four participants excluded due to poor quality of DNA extracted from buccal cells). Additional exclusion criteria based on behavioral performance, which are typically applied in priming research, were also defined beforehand (Kiefer 2019). These exclusion criteria are important to identify participants, who were not able to properly perform the task or who consciously identify the masked prime. Nine participants showing extremely high/low mean RT across all conditions of the priming paradigms (more than 2 *standard deviations* from the *mean* of the whole sample) and one participant who had a mean ER of 48.13% across all trials of the masked priming paradigm (near chance performance) and four participants who were able to identify 65% or more (confidence interval of chance performance) of the masked primes in the prime identification task (described in the following) correctly were excluded from further analyses. The data of the remaining 155 (121 females) healthy Caucasian volunteers was analyzed. The analyses including the 14 individuals excluded due to poor behavioral performance or due to conscious masked prime identification were reported in the Supplemental Material. Basically, the results of the analyses of the larger sample were comparable with those in the smaller sample reported in the manuscript. If there were differences, we discuss them in the Supplementary Material. Mean age of the final sample was 22.26 (*SD* = 3.63). One hundred fifteen participants (74.2%) were students; 39 participants (25.2%) had a college or technical college degree, and one participant (0.6%) had no school-leaving qualification. The study was approved by the ethics committee at Ulm University, Ulm, Germany. All participants gave written informed consent before participating in this study.

### Affective Neuroscience Personality Scales

We used the German version of the ANPS (Reuter et al. 2017). The ANPS comprises 110 items assessing individual tendencies in six primary emotional systems called SEEKING, CARE, and PLAY (positive emotionality) and FEAR, ANGER, and SADNESS (negative emotionality). The primary emotion of LUST is not assessed due to potential negative carryover effects on the remaining items, if items on one’s own sexual behavior would be filled in. All items are answered on a four-point Likert scale ranging from strongly disagree (1) to strongly agree (4). The average scores for SADNESS ranged from a minimum of 1.71 to a maximum of 3.57 (*M* = 2.54, *SD* = 0.36). Internal consistency of the SADNESS scale was acceptable (*α* = .71, *n* = 155).

### Genotyping

Deoxyribonucleic acid (DNA) was extracted from buccal cells. The purification was conducted by means of the MagNA Pure 96 system using the MagNA Pure 96 DNA kit from Roche Diagnostics, Mannheim, Germany. Genotyping of the BDNF Val66Met (rs6265) polymorphism was implemented on a “matrix-assisted laser desorption ionization-time of flight” (MALDI-TOF) platform (Agena; Massarray 4) by Varionostic, Ulm, Germany. Since 7 (4.5%) participants were homozygous for 66Met, 50 (32.3%) participants were heterozygous Val/Met carriers and 98 (63.2%) participants were Val66 homozygotes; Met/Met and Val/Met were treated as one group (Met+). Of note, the genotype distribution was in HWE: chi^2^ = 0.04, *p* = .85. There was no significant sex difference between the genotype groups (Val/Val 19 males, 79 females; Met+ 15 males, 42 females; *chi-squared*(1) = 1.01; *p* = .32). Even though genotype groups differed significantly in age (Val/Val: *M* = 22.72; *SD* = 4.22; Met+: *M* = 21.47, *SD* = 2.09; *t*(149.86) = 2.46; *p* < .05), age was not significantly correlated with any variable other than genotype neither for the whole sample nor when examining the genotype groups separately with two exceptions: In the Val/Val group, there was a significant association between age and the differences in RT of non-associated and indirectly associated trials in the unmasked priming paradigm (*r* = .21; *p* < .05), and in the Met+ group, there was a significant correlation of age and the difference in RT for the masked priming paradigm (*r* = − .37; *p* < .01). Therefore, we included age as covariate in all analyses examining group differences for BDNF genotype in these two variables.

### Assessment of Working Memory and EF

We assessed the capacity of verbal working memory as well as the ability to manipulate stored information using two digit span tasks adapted from the “Hamburg Wechsler Intelligenztest für Erwachsene” (HAWIE-R) (Tewes 1994). Working memory capacity was defined as the maximum number of digits the participant was able to recall correctly (range three to nine digits). The ability to manipulate stored information was assessed in a second digit span task and defined as the maximum number of digits the participant was able to recall correctly in reverse order (range two to eight digits). Any digit sequence of a given length had to be recalled twice (in both tasks). The maximum digit span was reached if the participant gave two wrong answers for both digit sequences of the same length. In the digit span forward task, participants recalled a minimum of three and a maximum of eight digits (*M* = 5.82, *SD* = 1.07). In the digit span backward task, participants recalled a minimum of two and a maximum of eight digits (*M* = 4.32, *SD* = 1.09).

### Procedure

After completing a German version of the “Edinburgh Handedness Inventory” (Oldfield 1971) identifying the hand which was used to respond, the main experiment started. Priming was assessed using a lexical decision task requiring the participant to decide if the target was a real word or a pseudoword. Pseudowords were defined as units of text appearing to be actual German words while not being lexically meaningful (e.g., “Nempen”) and were distractors. Primes were always German words. The participants were instructed to answer as quickly and accurately as possible. The answers were given by pressing one of two keys on a response box. The key for “word” was pressed with the index finger whereas the key pressed with the middle finger coded for the answer “pseudoword.” Depending on the participant’s handedness, the keys were laterally reversed. RT for distractor trials was not analyzed. All participants concluded the masked priming paradigm before being presented with the unmasked priming paradigm. Subjects performed 24 training trials at the beginning of each priming experiment. The experiments were programmed and presented by means of the software ERTS (Experimental Run Time System, Berisoft, Frankfurt, Germany).

The masked priming paradigm consisting of 160 trials (80 word-word and 80 word-pseudoword pairs) was adapted from previous experiments (Kiefer 2002; Kiefer and Brendel 2006; Reuter et al. 2009). Forty of the word-word pairs were directly (hen-egg) and the other 40 non-related pairs (leaf-car). Targets of the related conditions were matched for word length and word frequency (Kiefer 2002). Each trial started with a fixation cross for 750 ms followed by a mask consisting of nine random letters shown for 100 ms. Thereafter, the prime was presented for 33.5 ms followed by another random letter mask for 33.5 ms. Finally, the target stimulus, a German word or a pseudoword, was presented until the participant made a decision. Participants were not informed of the prime shown between the two masks.

The unmasked priming paradigm was adapted from Kiefer et al. (2005)) and consisted of 108 trials: 54 word-word and 54 word-pseudoword pairs. The word set of the unmasked priming paradigm was different from the masked priming paradigm. The word-word pairs consisted of 18 directly (hen-egg), 18 indirectly (lemon-sweet), and 18 non-related (leaf-car) pairs. Targets of the different semantic relatedness conditions were matched for word length and word frequency. Each trial started with a fixation cross for 700 ms before the prime was presented for 200 ms, followed immediately by the target shown until an answer was given. Descriptive statistics of the priming paradigm are presented in Table 1. Trials with RT ± 2 SD from an individual’s mean RT across all trials were excluded from analysis.

**Table 1:** Descriptive statistics of the unmasked and the masked priming paradigm

	*n*	*Minimum*	*Maximum*	*Mean*	*SD*
Unmasked					
RT (ms)					
Related	155	417.16	704.62	536.59	67.26
Indirectly related	155	444.49	756.19	571.99	67.01
Non-related	155	442.51	817.05	586.68	75.42
ER (%)					
Related	155	0.00	22.22	0.90	2.57
Indirectly related	155	0.00	27.78	2.76	4.45
Non-related	155	0.00	27.78	5.73	5.82
Masked					
RT (ms)					
Related	155	458.45	713.65	564.80	55.56
Non-related	155	453.97	729.97	584.41	59.02
ER (%)					
Related	155	0.00	22.50	1.89	2.92
Non-related	155	0.00	15.00	3.31	3.32

*RT* reaction time, *ER* error rate

After the experiment, participants were debriefed on the masked priming procedure, i.e., the existence of a prime between the forward and backward masks. The debriefing was followed by a recognition task in order to evaluate if the participants had been able to spot the prime in the masked condition. Therefore, 80 trials of the masked priming paradigm, 40 word-word and 40 word-letter string pairs, were presented while the participants were instructed to decide as accurately as possible whether the prime was a real word or a letter string comprising the repetition of the same capital letter (i.e., KKKKKK). In case participants were unable to identify the prime, they were instructed to guess. Right before the recognition task, five training trials were presented to ensure the participants’ understanding of the task. In order to analyze an individual’s detection accuracy independent of the response criterion, we calculated the sensitivity index *d′*. Therefore, we determined the relative frequencies of hits and false alarms for each participant and calculated *d*′ after applying *z* transformation (*d′* = *z*(hits) − *z*(false alarms)) (Green and Swets 1966). In order to rule out the possibility of backward priming effects from the target to the prime potentially facilitating the identification of the masked primes, we calculated *d′* sensitivity measures for the semantically related and unrelated conditions, respectively. We analyzed whether the sensitivity indices differed significantly from zero.

### Design and Statistical Analysis

Statistical analyses were conducted using IBM SPSS Statistics (version 24, IBM, USA) as well as the PROCESS macro (Hayes and Little 2017) for performing all multiple regression analyses. To test whether the BDNF Val66Met polymorphism is associated with semantic priming, repeated-measure ANOVAs were conducted in order to test the polymorphism’s associations with masked and unmasked semantic priming, respectively. The between-subject factor of the repeated-measure ANOVAs was genotype and had two levels (Met+ and Val/Val) whereas semantic relatedness as within-subject factor had two levels in the masked condition (direct or no semantic relatedness of prime and target) and three levels in the unmasked priming paradigm (direct, indirect, or no semantic relatedness of prime and target) resulting in a 2 × 2 or 2 × 3 mixed design, respectively. Dependent variables were the mean reaction time (RT) or mean error rate (ER). All significant effects of the repeated-measure ANOVAs are reported with Greenhouse-Geisser correction when appropriate. In order to evaluate which conditions differed significantly from each other, we conducted Tukey HSD post hoc analyses. Subsequently, we tested whether SADNESS or the scores of the digit span tasks differed as a function of BDNF Val66Met genotype using independent sample *t* tests. In order to analyze the associations of SADNESS, digit span tasks, and priming effects (differences between associated and non-associated trials), we calculated Pearson’s correlation coefficients. All *p* values of the reported correlation coefficients were Benjamini-Hochberg corrected for controlling false discovery rate (Benjamini and Hochberg 2000). In order to test if the association between BDNF Val66Met and semantic priming was mediated by performance in the digit span backward task, we planned on performing six multiple linear regression analyses including the independent variables BDNF Val66Met genotype and digit span with the dependent variable priming effects (two regression analyses for the masked priming paradigm (reaction times and error rates) and four (taking into account the indirectly related condition) regression analyses for the unmasked priming paradigm). The indirect effect of BDNF genotype on semantic priming would have been tested via bootstrapping using the PROCESS macro (Hayes and Little 2017). The mediation analyses could, however, not be performed (see the results). To test the moderation of the association between BDNF Val66Met genotype and EF as well as masked semantic priming by means of SADNESS, we performed four multiple linear regression analyses (one moderation analysis each with the dependent variable digit span forward as well as backward and one moderation analysis each with the dependent variable priming effect considering reaction times as well as error rates). Independent variables for all analyses were BDNF Val66Met genotype and SADNESS. The interaction of BDNF Val66Met genotype and SADNESS was introduced as an additional independent variable in a second step. We also explored the moderation of the association between BDNF Val66Met genotype and unmasked semantic priming by means of SADNESS. Since there were no significant results considering the moderation analyses in the unmasked priming paradigm, in the interest of clarity, the results of these analyses can be found in the Supplementary Material. Dependent variables were the scores of the digit span tasks and the differences in RT as well as ER (non-associated-associated). In order to avoid multicollinearity and potentially problematic effects with the interaction term, all variables in multiple regression analyses were centered. Only thereafter, the interaction term of BDNF Val66Met genotype and SADNESS was calculated (Aiken et al. 1991).

## Results

### Masked Prime Identification Task

Mean accuracy in the masked prime identification task was 50.85% (*SD* = 5.55%) and did not significantly differ from the chance level of 50% (*t*(154) = 1.92, *p* = .06). Mean accuracy did not significantly differ from chance neither for Val/Val homozygotes (*M* = 51.05%, SD = 5.77%, *t*(97) = 1.80, *p* = .08) nor for Met carriers (*M* = 50.53%, *SD* = 5.19%, *t*(56) = 0.78, *p* = .45). Further, mean accuracy did not significantly differ across genotypes (*t*(153) = 0.56, *p* = .58).

Neither *d′* for all (*M* = 0.05, *SD* = 0.33, *t*(154) = 1.86, *p* = .06) nor *d′* for semantically related (*M* = 0.04, *SD* = 0.39, *t*(154) = 1.36, *p* = .18) or non-related trials (*M* = 0.05, *SD* = 0.38, *t*(154) = 1.73, *p* = .09) did significantly differ from zero. Moreover, there was no significant influence of genotype on sensitivity indices, neither for general *d′* (Val/Val: *M* = 0.06, *SD* = 0.33; Met+: *M* = 0.02, *SD* = 0.31; *t*(153) = 0.78, *p* = .44) nor for *d′* in semantically related (Val/Val: *M* = 0.07, *SD* = 0.39; Met+: *M* = 0.00, *SD* = 0.38; *t*(153) = 1.04, *p* = .30) or for *d′* in semantically non-related trials (Val/Val: *M* = 0.06, *SD* = 0.40; Met+: *M* = 0.04, *SD* = 0.37; *t*(153) = 0.33, *p* = .75). Thus, it can be concluded that the masked primes were invisible in all participant groups.

### Semantic Priming and BDNF Val66Met

The repeated-measure ANOVAs with RT and ER as dependent variables, semantic relatedness as within and BDNF Val66Met genotype as between-subject factor revealed significant priming effects for masked and unmasked priming paradigms (Table 2). Participants showed reduced RT and ER, when prime and target were semantically related as compared with trials in which prime and target were not semantically related. Even indirectly related prime target pairs resulted in a facilitation of performance compared with semantically non-related prime-target pairings.

**Table 2:** Priming effects for the masked and unmasked priming paradigm

Behavioral data	Related (A)	Indirectly related (B)	Non-related (C)	Repeated-measure ANOVA	Post hoc (Tukey HSD)
Unmasked					
RT (ms)	*M* = 536.59	571.99	586.68	*F*(2, 304) = 9.49	A < B
	*SE* = 5.40	5.38	6.06	*p* < .001	A < C
Covariate: age					B < C
ER (%)	*M* = 0.90	2.76	5.73	*F*(1.67, 255.88) = 52.19	A < B
	*SE* = 0.21	0.36	0.47	*p* < .001	A < C
					B < C
Masked					
RT (ms)	*M* = 564.80	–	584.41	*F*(1, 152) = 11.42	A < C
	*SE* = 4.46	–	4.74	*p* < .01	
Covariate: age					
ER (%)	*M* = 1.89	–	3.31	*F*(1, 153) = 30.51	A < C
	*SE* = 0.23	–	0.27	*p* < .001	
					

*M* mean, *SE* standard error of the mean,  partial eta-squared, *RT* reaction time, *ER* error rate

Looking at RT in the unmasked priming paradigm, there was neither a significant main effect of genotype (*F*_(1, 152)_ = 0.07; *p* = .79) nor a significant interaction of genotype and semantic relatedness (*F*_(2, 304)_ = 0.59; *p* = .55). The same result pattern was obtained when analyzing the ER. There was no significant main effect of BDNF genotype (*F*_(1, 153)_ = 3.39; *p* = .07) and no significant interaction of BDNF Val66Met and semantic relatedness (*F*_(1.67, 255.88)_ = 1.03 *p* = .35). It is, however, worth noting that on a descriptive level, Met+ individuals made more errors especially in semantically indirectly and non-related trials (Val/Val: related: *M* = 0.79, *SD* = 1.95; indirectly related: *M* = 2.15, *SD* = 3.35; non-related: *M* = 5.44, *SD* = 5.64; Met+: related: *M* = 1.07, *SD* = 3.39; indirectly related: *M* = 3.80, *SD* = 5.77; non-related: *M* = 6.24, *SD* = 6.13; all percentages).

**Fig. 1:**
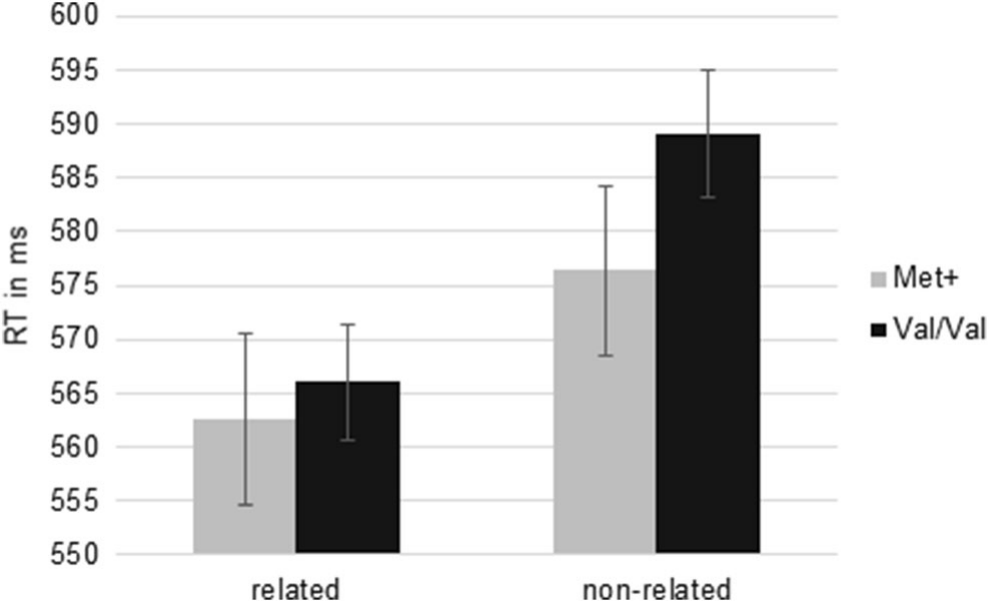
Mean RT of lexical decisions in the masked priming paradigm. Whiskers show *standard errors of the mean* (*SEs*). Met+ individuals showed significantly less RT priming as compared with Val/Val homozygotes

**Fig. 2:**
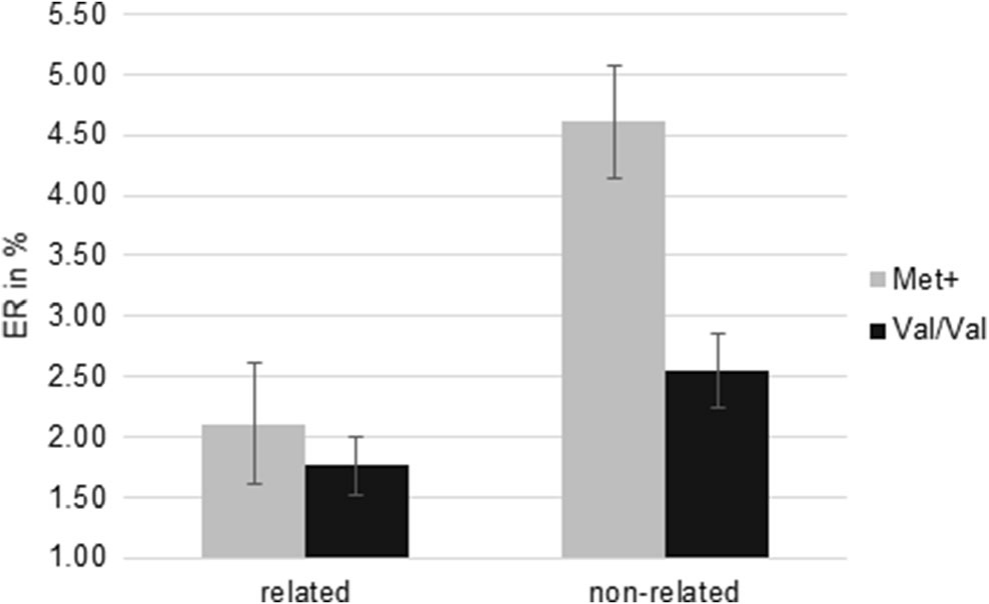
Mean error rate (ER) of lexical decisions in the masked priming paradigm. Whiskers show *SEs*. Met+ individuals showed significantly higher error rates in non-related trials than Val/Val homozygotes

In the masked priming paradigm, there was no significant main effect of genotype (*F*_(1, 152)_ = 0.56; *p* = .46) but a significant interaction between BDNF Val66Met and semantic relatedness (*F*_(1, 152)_ = 8.14; *p* < .01;  = .05; Fig. 1). Tukey HSD post hoc analyses (Table 3) revealed that both Met+ as well as Val/Val carriers showed significant priming effects as indicated by significantly faster RTs comparing semantically related with semantically non-related trials (Val/Val: *p* < .001; Met+: *p* < .001). There were no significant differences between semantically related or semantically non-related prime target pairings comparing Val/Val and Met+ individuals. The significant interaction originates from smaller priming effects in Met+ individuals as compared with Val/Val homozygotes (Fig. 1). Conversely, analyzing ER revealed a significant main effect of BDNF Val66Met genotype (*F*_(1, 153)_ = 8.44; *p* < .01;  = .05). Met+ individuals made more errors across all trials (*M* = 4.10; *SD* = 3.54) than Val66 homozygotes (*M* = 2.98; *SD* = 2.30). Furthermore, the interaction between semantic relatedness and BDNF Val66Met genotype was significant (*F*_(1, 153)_ = 8.23; *p* < .01;  = .05, Fig. 2). Met+ individuals made significantly more errors in non-related trials than in related trials, while Val/Val homozygotes did not show significant priming effects as indicated by the non-significant difference of ER in semantically related and non-related trials (Fig. 2). Moreover, Met+ individuals’ error rates in non-related trials were significantly higher than Val/Val homozygotes’ error rates irrespective of semantic relatedness (Table 3).

**Table 3:** Post hoc analyses for the interaction term in the masked priming paradigm

	BDNF Val66Met	Semantic relatedness	*M* (*SE*) (ms)	Post hoc (Tukey HSD)	1	2	3
RT							
1	Val/Val	Related	566.06 (5.38)				
2	Val/Val	Non-related	589.08 (5.93)	*p*	< .001		
3	Met+	Related	562.62 (7.90)	*p*	.98	.03	
4	Met+	Non-related	576.38 (7.84)	*p*	.70	.54	< .001
ER							
1	Val/Val	Related	1.76 (0.24)				
2	Val/Val	Non-related	2.55 (0.30)	*p*	.13		
3	Met+	Related	2.11 (0.50)	*p*	.91	.82	
4	Met+	Non-related	4.61 (0.47)	*p*	< .001	< .001	< .001

*M* mean, *SE* standard error of the mean, *RT* reaction time, *ER* error rate

### Group Differences in SADNESS Scores and in Digit Span Performance as a Function of BDNF Val66Met Genotype

Neither SADNESS scores nor digit span performance differed between Val/Val and Met+ individuals (Table 4). Since BDNF Val66Met genotype was not associated with digit span performance, the intended mediation analysis of EF (as measured by digit span performance) mediating the association between BDNF Val66Met genotype and semantic priming could not be performed.

**Table 4:** Descriptive statistics and independent sample *t* tests comparing genotype groups for SADNESS and EF

	BDNF Val66Met	*n*	*M*	*SD*	*t*	*df*	*p*
SADNESS	Val/Val	98	2.52	0.37	− 0.81	153	.42
	Met+	57	2.57	0.34			
Digit span(f)	Val/Val	98	5.71	1.08	− 1.62	153	.11
	Met+	57	6.00	1.02			
Digit span(b)	Val/Val	98	4.20	1.06	− 1.69	153	.09
	Met+	57	4.51	1.14			

*f* forward, *b* backward

### Correlational Analysis of SADNESS, EF as well as Masked and Unmasked Priming Effects

Contradictory to our expectations, SADNESS scores were not significantly associated with any of the examined variables (Table 5). The score of the digit span forward task was only significantly associated with the score of the digit span backward task. In case of the digit span backward task being a stronger representative of EF than the score of the digit span forward task, we only found a significant positive association with the difference in ER priming effects in the masked priming paradigm.

**Table 5:** Pearson correlation coefficients between SADNESS, digit span tasks, and priming effects

		SADNESS	Digit span(f)	Digit span(b)
Digit span(f)	*r*	− .04		
Digit span(b)	*r*	.07	.37***	
Unmasked RT (N-A)	*r*	.07	− .01	.04
Unmasked RT (N-I)	*r*	.11	− .04	.04
Unmasked ER (N-A)	*r*	.01	.02	− .01
Unmasked ER (N-I)	*r*	.01	.00	.03
Masked RT (N-A)	*r*	.01	− .13	− .11
Masked ER (N-A)	*r*	− .08	.15	.24*

All *p* values were Benjamini-Hochberg corrected for false discovery rate

*f* forward, *b* backward, *RT* reaction times, *ER* error rate

***p < .001; *p < .05

### Moderation of the Association Between BDNF Val66Met Genotype and Performance in Digit Span Tasks by SADNESS Scores

Regression analyses with performance in the digit span forward task as dependent variable and the independent variables BDNF Val66Met genotype and SADNESS did not explain a significant amount of variance (*R*^2^ = .01, *F*_(2, 152)_ = 1.46, *p* = .24). The additional inclusion of the interaction term did not result in a significant increment in explained variance (*R*^2^ = .01, *F*_(3, 151)_ = 0.97, *p* = .41).

**Fig. 3:**
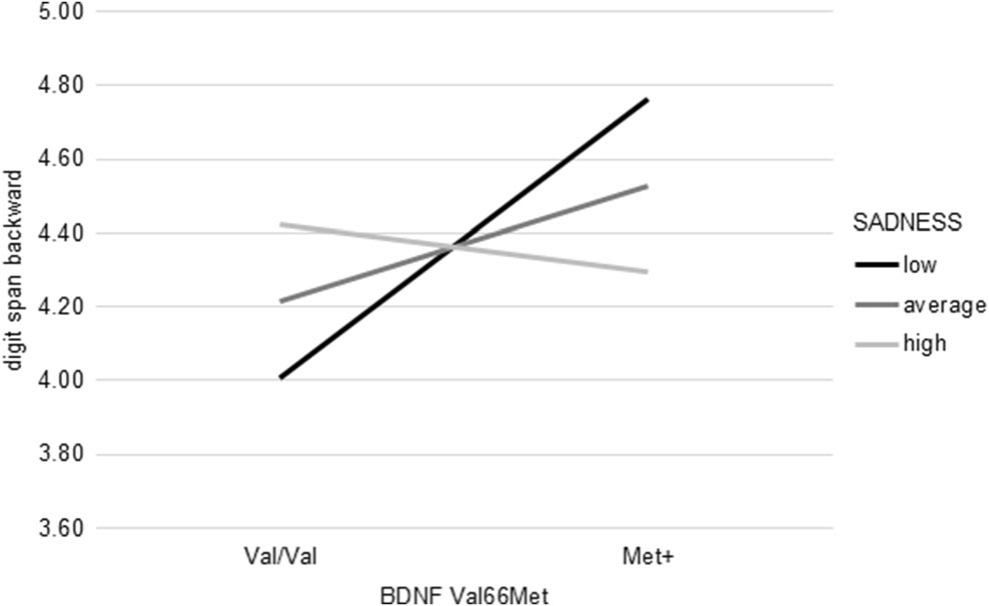
SADNESS significantly moderated the association between BDNF Val66Met genotype and the performance in the digit span backward task. High/low refers to mean ± 1 *SD*

In the multiple regression analyses with performance in the digit span backward task as dependent variable, BDNF Val66Met genotype and SADNESS alone did not explain a significant amount of variance (*R*^2^ = .22, *F*_(2, 152)_ = 1.70, *p* = .19); introduction of the interaction term, however, yielded a significant increase in explained variance (*R*^2^ = .24, *F*_(3, 151)_ = 3.05, *p* < .05, *b* = − 1.23, *t*(151) = − 2.38, *p* < .05). Low SADNESS scores were associated with worse performance in the digit span backward task for Val/Val homozygotes while Met+ individuals showed improved performance in case of low SADNESS scores. Further, differences between Val/Val homozygotes and Met+ individuals declined with increasing SADNESS scores (Fig. 3).

### Moderation of the Association Between BDNF Val66Met Genotype and Differences in Masked Priming by SADNESS Scores

**Fig. 4:**
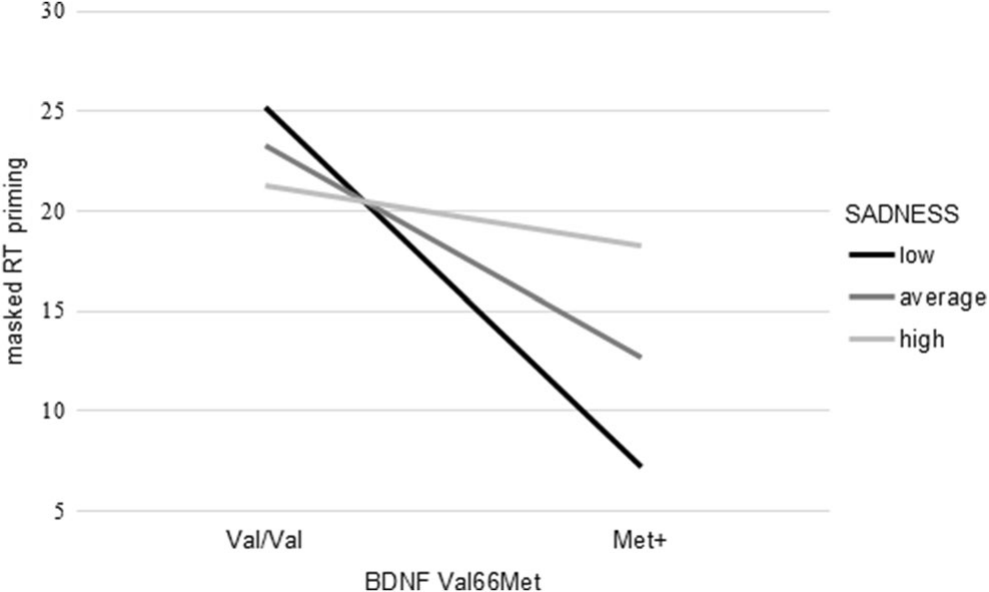
SADNESS significantly moderated the association between BDNF Val66Met genotype and masked RT priming effects. High/low refers to mean ± 1 *SD*. Estimates are based on setting the covariate to its sample mean. Masked RT priming corresponds to differences in reaction times (in ms) between trials with semantically related and trials with semantically non-related prime-target pairings

In the moderation analyses with masked RT priming as dependent variable, BDNF Val66Met genotype and SADNESS together with the covariate age explained a significant amount of variance (*R*^2^ = .06, *F*_(3, 151)_ = 3.22, *p* < .05). Further, the introduction of the interaction term yielded a significant increase in explained variance (∆*R*^2^ = .03, *F*_(4, 150)_ = 3.51, *p* < .01, *b* = 20.89, *t*(150) = 2.04, *p* < .05). Low SADNESS scores were associated with larger priming effects in Val/Val homozygotes while Met+ carriers showed almost no priming in case of low SADNESS scores. As for performance in the digit span backward task, differences between Val/Val homozygotes and Met+ individuals declined with increasing SADNESS scores. Additionally, SADNESS showed a stronger influence in Met+ individuals as compared with Val/Val homozygotes (Fig. 4).

**Fig. 5:**
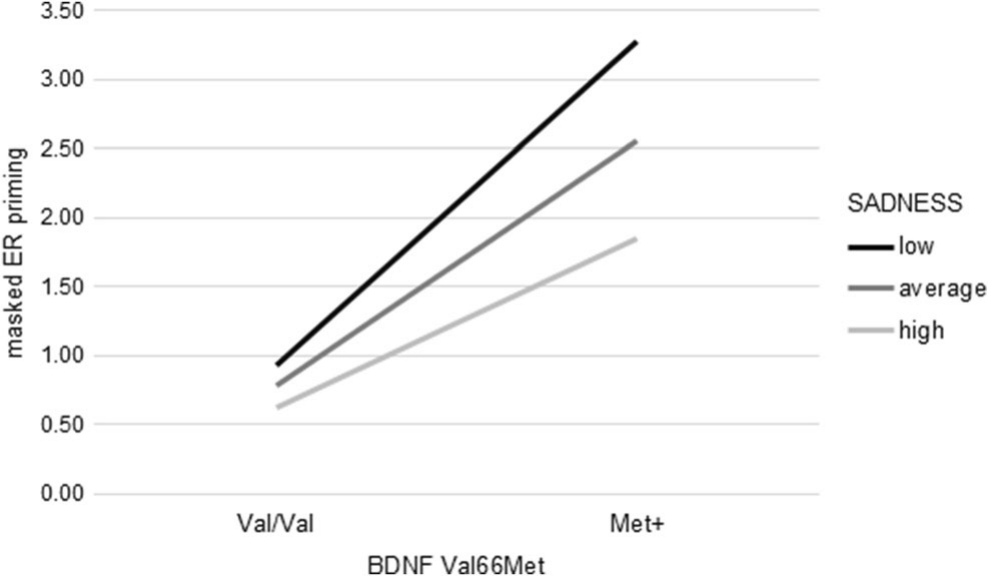
SADNESS did not moderate the association between BDNF Val66Met genotype and masked ER priming effects. Masked ER priming refers to differences in error rates (in %) between trials with semantically related and trials with semantically non-related prime-target pairings. High/low refers to mean ± 1 *SD*

In the regression analyses with masked ER priming as dependent variable, the independent variables BDNF Val66Met genotype and SADNESS explained a significant amount of variance (*R*^2^ = .06, *F*_(2, 152)_ = 4.78, *p* < .01). However, the additional inclusion of the interaction term did not result in a significant increment in explained variance (∆*R*^2^ = .01, *F*_(3, 151)_ = 3.46, *p* < .05, *b* = − 1.55, *t*(151) = − 0.90, *p* = .37; Fig. 5).

## Discussion

The present study aimed to investigate the associations between the BDNF Val66Met polymorphism, EF, depressive mood, and semantic priming. Building on previous findings and theorizing (Beste et al. 2011; Egan et al. 2003; Kiefer et al. 2005), we assumed the 66Met allele to be associated with impaired working memory and larger priming effects. Further, basing on previous findings showing interactions of BDNF Val66Met genotype and stress as well as depressed mood in predicting cognitive functions (Beste et al. 2011; Gatt et al. 2009), we expected the SADNESS scale of the ANPS measuring depressive emotionality to be a moderator of the associations of BDNF Val66Met and working memory as well as semantic priming.

We found the BDNF Val66Met polymorphism to be associated with masked priming, but not with unmasked priming. However, the priming pattern was not congruent with our prediction: Met+ individuals showed smaller masked RT priming effects, but larger ER priming effects. ER priming effects were positively related to performance in the digit span backward task. Moreover and most importantly, as expected, SADNESS moderated the association between BDNF Val66Met and the digit span backward task as well as the association between BDNF Val66Met and RT priming in the masked priming paradigm.

### Executive Functions

Contrary to our expectations, Met+ individuals did not perform poorer in the digit span tasks. In fact, digit span performance was comparable for Met+ and Val/Val groups. However, we found a moderation of the association between BDNF Val66Met and the digit span backward task by the primary emotion SADNESS. In case of low SADNESS scores, the 66Met allele was related to increased digit span backward performance.

Possibly, Met+ individuals have superior EF provided that they are not exposed to stressful life events or other circumstances associated with negative mood. This interpretation is in line with a previous study (Gatt et al. 2009) reporting a similar moderation. In conditions with low early life stress, Met+ individuals showed improved working memory performance, higher hippocampal gray matter volume, and lower heart rate compared with Val/Val homozygotes. Another study reported Met+ individuals exposed to adverse childhood events to demonstrate a lower proportion of positive recall in a self-referent encoding task than Val/Val homozygotes (van Oostrom et al. 2012). These studies in combination with our results indicate Met+ individuals to be high functioning but also more vulnerable and less resilient to the influences of stressful life events and negative emotionality. This assumption contradicts studies suggesting the 66Met allele to be associated with impaired cognitive functioning independent of stressful life events or depressive emotionality (Beste et al. 2011; Egan et al. 2003; Montag et al. 2014). However, if carrying the 66Met allele would solely have negative consequences for the individual (like an increased risk of developing major depression or impaired cognitive functioning), the question would arise as to why the 66Met allele was evolutionarily preserved, even constituting the predominant allele in Asian countries with a frequency of up to 60% (www.ncbi.nlm.nih.gov).

In contrast to the assumed increased vulnerability of Met+ individuals to the influences of negative mood or life stress, a previous study found iconic memory performance to be positively correlated with depressed mood in Val/Val homozygotes only (Beste et al. 2011). It is, however, worth mentioning that the non-significant correlation of iconic memory performance and depressed mood in Met+ individuals could have been a result of generally low iconic memory performance of Met+ individuals. Moreover, the sample consisted solely of individuals suffering from subclinical or mild depressive symptom severity potentially causing an underestimation of the interaction due to limited variances (Beste et al. 2011).

The non-significant but negative association between performance in the digit span backward task and masked RT priming is in line with our initial hypothesis and with previous research showing a negative correlation of EF and semantic priming (Kiefer et al. 2005). Since this correlation did not reach significance in the present study, our data does not support an influence of EF on semantic priming regarding RT. The study by Kiefer et al. (2005), however, examined a sample comprising participants of all ages and levels of education. In the present study, we examined students yielding a limited variance in EF, which might have diminished the association between EF and RT priming. In contrast, there was a significantly positive association between performance in the digit span backward task and ER priming. This result pattern indicates high EF to influence RT priming in the opposite direction as compared with ER priming. Possibly, high EF individuals use a more superficial processing style of the lexical decision target. Since a similar RT and ER priming pattern was also observed in Met+ carriers, we discuss this issue in more detail in the following section.

### BDNF Val66Met and Semantic Priming

In the masked priming paradigm, there was a significant interaction between genotype and semantic relatedness for both RT and ER data. For RT, Met+ individuals had smaller priming effects than Val/Val homozygotes. For ER, Met+ individuals had larger priming effects than the Val/Val group. This larger ER priming effect was mainly driven by the high ER of Met+ individuals in semantically non-related trials. Val/Val homozygotes on the other hand did not show significant differences in ER between trials with semantically related and semantically non-related prime-target pairings.

In contrast to our expectations, Met+ individuals did not show greater RT and ER priming (related to differences in working memory capacity) compared with Val/Val homozygotes. Instead, magnitude of RT and ER priming was reversed in these individuals with smaller RT and higher ER priming. This complex priming pattern most likely indicates a faster and more superficial processing style in Met+ individuals compared with Val/Val homozygotes: Met+ individuals responded rapidly to the lexical decision targets, possibly based on their focus on the target’s visual word form without in-depth analysis of its lexical status. In addition to generally faster reactions, this processing style focused on word form might have led to reduced RT priming by diminishing the influence of prime processing on response speed. At the same time, this fast processing style focused on visual word form seems to increase the likelihood for erroneous pseudoword responses to word targets in general, but specifically for semantically non-related prime-target pairings. Perhaps, in the non-related condition, in which the target representation is not pre-activated, fluency of target processing is diminished. This diminished target fluency of word targets in the non-related condition might specifically bias Met+ individuals to provide an erroneous pseudoword response due to their fast and superficial processing style. There was neither a main effect for BDNF Val66Met genotype nor an interaction between BDNF Val66Met and semantic relatedness on RTs or ERs in the unmasked priming paradigm. Possibly, visibility of the primes in the unmasked conditions induces the application of strategic priming mechanisms such as postlexical semantic matching (Neely et al. 1989) in either BDNF Val66Met genotype group so that spontaneous differences in habitual processing styles disappear.

SADNESS significantly moderated the association between BDNF Val66Met and masked semantic RT priming. While low SADNESS Met+ individuals showed smallest priming effects, priming effects observed for Met+ individuals and Val/Val homozygotes were more similar with increasing SADNESS. Furthermore, Met+ individuals showed a non-significant but strong decline in ER priming as compared with Val/Val homozygotes. Thus, the aforementioned vulnerability to depressed emotionality in Met+ individuals might not only affect EF, but also influence their processing style in the lexical decision task. Low SADNESS Met+ individuals did not only display high performance in the digit span backward task, but also showed smallest RT and largest ER priming effects indicative of a fast and superficial processing style. For high SADNESS genotype differences in RT and ER priming decrease between Met+ and Val/Val individuals, possibly indicating a less superficial processing style.

As we did not assess the neural correlates of priming with neuroimaging techniques, we cannot specify differences in brain structure and function between BDNF Val66Met genotype groups in our study. Given the known functional neuroanatomy of semantic priming (Norman and Shallice 1986; Ulrich et al. 2013, 2014; Wagner et al. 2001), we speculate that functional neuroanatomical differences mainly concern prefrontal and temporal areas. It must of course also remain open whether the putative difference in processing styles between BDNF Val66Met genotype groups is the consequence of possible differences in functional neuroanatomy or vice versa. Finally, in the present study, effects of the BDNF Val66Met polymorphism were only observed in the digit span backward task and in the masked priming paradigm, but not in unmasked priming. Future studies could therefore assess altered cognitive functions in BDNF Val66Met genotype groups in general and altered processing styles in particular using a variety of cognitive tasks also in other domains than EF and semantic processing.

### Limitations and Future Directions

Some limitations need to be considered interpreting the results of the present study. First, despite our sample was large for an experimental study, unraveling the genetic underpinnings of human behavior might need even larger samples (Montag and Reuter 2014). This is especially important since the homozygous Met/Met variant of the BDNF Val66Met polymorphism only occurs in 2–3% of the Caucasian population (Shimizu et al. 2004) making a separate examination of homozygous Met+ individuals impossible in our study. Second, EF was measured using only the digit span backward task. Results across studies may become more consistent examining EF using additional tasks, for instances a Stroop or flanker task (Eriksen and Eriksen 1974; Stroop 1935). Additional testing could also enable a more sophisticated evaluation of which exact components of EF (updating, inhibition, or switching) are associated with BDNF Val66Met and semantic priming. Differential processing styles as a function of the BDNF Val66Met polymorphism should be investigated in other tasks than a primed lexical decision task. Third, we did only look at one polymorphism. Future studies should also investigate gene-gene interactions. There are for example indications for an interaction of BDNF Val66Met and 5-HTTLPR (serotonin transporter–linked polymorphic region), a polymorphism in the serotonin transporter gene, considering neural substrates related to sadness and EF (Wang et al. 2012). Furthermore, interactions with the DRD2/ANKK1 polymorphism have been reported (Montag et al. 2010b; Montag et al. 2010d; Walter et al. 2011). Last, we did not use imaging procedures. With respect to our results and the given explanations, neuroimaging would have been especially informative about how the BDNF Val66Met polymorphism is associated with neuronal activity of different brain areas and how these brain areas in turn influence semantic priming.

## Conclusion

In summary, this study for the first time revealed associations of BDNF Val66Met genotype and unconscious automatic processing assessed using a masked semantic priming paradigm. The BDNF Val66Met polymorphism explained a significant amount of variance in masked semantic priming. Met+ individuals showed reduced RT priming compared with Val/Val homozygotes and significantly higher ER priming resulting from high error rates in semantically non-related trials. Further, the polymorphism interacted with the primary emotion SADNESS influencing EF as well as unconscious semantic processing. We suggest that Met+ individuals with low depressive tendencies have not only superior EF, but also a faster and more superficial processing style, compared with Val/Val homozygotes. However, in Met+ individuals, cognitive functioning exhibits a greater vulnerability to depressed emotionality compared with Val/Val homozygotes. Our study thus demonstrates how emotional and molecular genetic factors exert an interacting influence on higher-level cognition.

## Electronic Supplementary Material

ESM 1 (DOCX 54 kb)
