# Supplementary material for: Depressive Emotionality Moderates the Influence of the BDNF Val66Met Polymorphism on Executive Functions and on Unconscious Semantic Priming
Source: J Mol Neurosci. 2020 Jan 30;70(5):699–712. doi: 10.1007/s12031-020-01479-x (PMC7152588; doi:10.1007/s12031-020-01479-x)
Supplement: Supplementary file 1 — (DOCX 54 kb) [file 12031_2020_1479_MOESM1_ESM.docx]

Depressive emotionality moderates the influence of the BDNF Val66Met polymorphism on executive functions and on unconscious semantic priming

Simon Sanwald^1^*, Christian Montag^2§^ and Markus Kiefer^1§^

^1^Ulm University, Department of Psychiatry, Ulm, Germany

^2^Ulm University, Department of Psychology, Ulm, Germany

§ Both authors contributed equally to this work

**Journal of Molecular Neuroscience**

*Correspondence to:

Simon Sanwald

Ulm University

Department of Psychiatry

Section for Cognitive Electrophysiology

Leimgrubenweg 12, 89075 Ulm, Germany

Phone: +49 731 500 61538 Fax: +49 731 500 61542

Email: [Simon.Sanwald@uni-ulm.de](mailto:Simon.Sanwald@uni-ulm.de)

# **Supplemental Material**

# *Identification task and BDNF Val66Met genotype*

Table S1.

Results of the identification task.

|  | BDNF Val66Met | *n* | *M* | *SD* | *t* | *df* | *p* |
| --- | --- | --- | --- | --- | --- | --- | --- |
| Mean accuracy | Val/Val | 98 | 51.05 | 5.77 | 0.56 | 153 | .58 |
|  | Met+ | 57 | 50.53 | 5.19 |  |  |  |
| *d‘* | Val/Val | 98 | 0.06 | 0.33 | 0.78 | 153 | .44 |
|  | Met+ | 57 | 0.02 | 0.31 |  |  |  |
| *d‘_A_* | Val/Val | 98 | 0.07 | 0.39 | 1.04 | 153 | .30 |
|  | Met+ | 57 | 0.00 | 0.38 |  |  |  |
| *d‘_N_* | Val/Val | 98 | 0.06 | 0.40 | 0.33 | 153 | .75 |
|  | Met+ | 57 | 0.04 | 0.37 |  |  |  |

# *Moderation of the association between BDNF Val66Met genotype and differences in performance in the lexical decision task for the unmasked semantic priming paradigm by means of SADNESS*

Moderation analyses for the unmasked priming paradigm only showed a significant moderation effect of SADNESS considering the association between BDNF Val66Met and differences in error rates (indirectly semantically related – semantically related).

*Differences in reaction times*

*Non-related – related*

The independent variables did not explain a significant amount of variance in the difference in reaction times in the lexical decision task of non-related and related trials (*R^2^* = .03, *F*(3,151) = 1.54, *p* = .21; Table S2).

Table S2.

Regression model of the moderation analysis for RTs

|  | *b* | *SE* | *t* | *p* |
| --- | --- | --- | --- | --- |
| Constant | 8.83 | 23.52 | 0.38 | .71 |
| SADNESS | 15.63 | 9.24 | 1.69 | .09 |
| BDNF Val66Met | 77.73 | 42.12 | 1.85 | .07 |
| SADNESS*BDNF | -28.55 | 16.35 | -1.75 | .08 |

*Indirectly related – related*

The independent variables did not explain a significant amount of variance in the difference in reaction times in the lexical decision task of indirectly related and related trials (*R^2^* = .04, *F*(4,150) = 1.44, *p* = .22; Table S3).

Table S3.

Regression model of the moderation analysis for RTs

|  | *b* | *SE* | *t* | *p* |
| --- | --- | --- | --- | --- |
| Constant | -51.82 | 29.02 | -1.79 | .08 |
| SADNESS | 14.05 | 9.28 | 1.51 | .13 |
| BDNF Val66Met | 36.26 | 42.33 | 0.86 | .39 |
| SADNESS*BDNF | -12.27 | 16.46 | -0.75 | .46 |
| Age | 1.31 | 0.77 | 1.70 | .09 |

*Differences in error rates*

*Non-related – related*

The independent variables did not explain a significant amount of variance in the difference in error rates in the lexical decision task of non-related and related trials (*R^2^* = .01, *F*(3,151) = 0.39, *p* = .76; Table S4).

Table S4.

Regression model of the moderation analysis for ERs

|  | *b* | *SE* | *t* | *p* |
| --- | --- | --- | --- | --- |
| Constant | 6.86 | 4.30 | 1.59 | .11 |
| SADNESS | -0.88 | 1.69 | -0.52 | .60 |
| BDNF Val66Met | -6.82 | 7.70 | -0.89 | .38 |
| SADNESS*BDNF | 2.88 | 2.99 | 0.96 | .34 |

*Indirectly related – related*

The independent variables did not explain a significant amount of variance in the difference in error rates in the lexical decision task of indirectly related and related trials (*R^2^* = .03, *F*(3,151) = 1.64, *p* = .18; Table S5). However, the interaction term was a significant predictor of the difference in error rates:

Table S5.

Regression model of the moderation analysis for ERs

|  | *b* | *SE* | *t* | *p* |
| --- | --- | --- | --- | --- |
| Constant | 7.99 | 4.54 | 1.76 | .08 |
| SADNESS | -1.87 | 1.78 | -1.05 | .30 |
| BDNF Val66Met | -17.55 | 8.13 | -2.16 | .03 |
| SADNESS*BDNF | 6.54 | 3.16 | 2.07 | .04 |

*Analyses with N = 169 participants*

We additionally performed all analyses of the present study without excluding participants who showed slow RTs, high ERs or masked prime recognition performance above chance level (conscious masked prime identification). We thereby want to explore how results of the current study change due to exclusion criteria. 107 individuals were Val/Val homozygotes and 62 Met+ carriers. Descriptive statistics can be found in Table S6. Results of the analyses in the larger sample were comparable to those in the smaller sample reported in the manuscript. Only the results of the moderation analyses changed. We discuss these changes in the appropriate section.

Table S6.

Descriptive statistics of the unmasked and masked priming paradigm (*N* = 169)

|  | *n* | *minimum* | *maximum* | *mean* | *sd* |
| --- | --- | --- | --- | --- | --- |
| *unmasked* |  |  |  |  |  |
| RT in ms |  |  |  |  |  |
| related | 169 | 417.16 | 981.83 | 547.08 | 84.62 |
| indirectly related | 169 | 444.49 | 980.92 | 582.40 | 85.57 |
| non- related | 169 | 442.51 | 1035.91 | 597.73 | 93.64 |
| ER in % |  |  |  |  |  |
| related | 169 | 0.00 | 22.22 | 0.95 | 2.78 |
| indirectly related | 169 | 0.00 | 27.78 | 2.63 | 4.37 |
| non- related | 169 | 0.00 | 27.78 | 5.49 | 5.72 |
| *masked* |  |  |  |  |  |
| RT in ms |  |  |  |  |  |
| related | 169 | 458.45 | 918.84 | 572.87 | 71.26 |
| non- related | 169 | 453.97 | 1029.32 | 593.85 | 77,68 |
| ER in % |  |  |  |  |  |
| related | 169 | 0.00 | 50.00 | 2.09 | 4.67 |
| non- related | 169 | 0.00 | 55.00 | 3.49 | 5.14 |

*Note.* RT = reaction time; ER = error rate.

*Repeated measures ANOVA*

Table S7.

Priming effects for the masked and unmasked priming paradigm

| behavioral data | related (A) | indirectly related (B) | non-related (C) | repeated-measures ANOVA | Post-hoc (*Tukey HSD*) |
| --- | --- | --- | --- | --- | --- |
| unmasked |  |  |  |  |  |
| RT | *M* = 547.08 | 582.40 | 597.73 | *F*(2,332) = 10.31 | A < B |
| in ms | *SD* = 84.62 | 85.57 | 93.64 | *p* < .001 | A < C |
| covariate: age |  |  |  | $\text{ƞ}_{\text{p}}^{\text{2}}$= .06 | B < C |
| ER | *M* =0.95 | 2.63 | 5.49 | *F*(1.67,279.51) = 51.34 | A < B |
| in % | *SD* =2.78 | 4.37 | 5.72 | *p* < .001 | A < C |
|  |  |  |  | $\text{ƞ}_{\text{p}}^{\text{2}}$= .24 | B < C |
| masked |  |  |  |  |  |
| RT | *M* = 572.87 | - | 593.85 | *F*(1,166) = 6.48 | A < C |
| in ms | *SD* = 71.26 | - | 77,68 | *p* = .01 |  |
| covariate: age |  |  |  | $\text{ƞ}_{\text{p}}^{\text{2}}$= .04 |  |
| ER | *M* = 2.09 | - | 3.49 | *F*(1,167) = 33.43 | A < C |
| in % | *SD* = 4.67 | - | 5.14 | *p* < .001 |  |
|  |  |  |  | $\text{ƞ}_{\text{p}}^{\text{2}}$= .17 |  |

*Note.* *M* = mean; *SE* = standard error of the mean; $\text{ƞ}_{\text{p}}^{\text{2}}$= partial eta squared; RT = reaction time; ER = error rate.

Looking at RT in the unmasked priming paradigm (for effects of semantic relatedness see Table S7) there was neither a significant main effect of genotype (*F*(1,166) = 0.60; *p* = .44) nor a significant interaction of genotype and semantic relatedness (*F*(2,332) = 0.81; *p* = .44). The same result pattern was obtained when analyzing the ER. There was no significant main effect of BDNF genotype (*F*(1,167) = 2.45; *p* = .12) and no significant interaction of BDNF Val66Met and semantic relatedness (*F*(1.67,279.51) = 0.83; *p* = .42).

In the masked priming paradigm there was no significant main effect of genotype (*F*(1,166) = 0.73; *p* = .40) but a significant interaction between BDNF Val66Met and semantic relatedness (*F*(1,166) = 4.81; *p* = .03; $\text{ƞ}_{\text{p}}^{\text{2}}$= .03). Tukey HSD post-hoc analyses (Table S8) revealed that both Met+ as well as Val/Val carriers showed significant priming effects as indicated by significantly faster RTs comparing semantically related to semantically non-related trials (Val/Val: *p* < .001; Met+: *p* < .001). There were no significant differences between semantically related or semantically non-related prime target pairings comparing Val/Val and Met+ individuals. The significant interaction originates from smaller priming effects in Met+ individuals as compared to Val/Val homozygotes.

Conversely, analyzing ER revealed a significant main effect of BDNF Val66Met genotype (*F*(1,153) = 8.44; *p* < .01; $\text{ƞ}_{\text{p}}^{\text{2}}$= .05). Met+ individuals made more errors across all trials (*M* = 4.10; *SD* = 3.54) than Val66 homozygotes (*M* = 2.98; *SD* = 2.30). Furthermore, the interaction between semantic relatedness and BDNF Val66Met genotype was significant (*F*(1,153) = 8.23; *p* < .01; $\text{ƞ}_{\text{p}}^{\text{2}}$= .05). Tukey HSD post-hoc analyses revealed that both Met+ as well as Val/Val carriers showed significant priming effects as indicated by significantly lower ERs comparing semantically related to semantically non-related trials (Val/Val: *p* < .05; Met+: *p* < .001). Moreover, Met+ individuals’ ERs in non-related trials were high, accounting for the stronger priming effect in these individuals as compared to Val/Val homozygotes.

Table S8.

Post-hoc analyses for the interaction term in the masked priming paradigm

| RT | | | | | | | |
| --- | --- | --- | --- | --- | --- | --- | --- |
|  | BDNF Val66Met | semantic relatedness | *M (SE)* in ms | Post-hoc (*Tukey HSD*) | 1 | 2 | 3 |
| 1 | Val/Val | related | 568.80 (5.41) |  |  |  |  |
| 2 | Val/Val | non-related | 592.70 (6.05) | *p* | **< .001** |  |  |
| 3 | Met+ | related | 579.88 (11.69) | *p* | .78 | .70 |  |
| 4 | Met+ | non-related | 595.83 (12.58) | *p* | .11 | .99 | **< .001** |
| ER | | | | | | | |
|  | BDNF Val66Met | semantic relatedness | *M (SE)* in % | Post-hoc *(Tukey HSD)* | 1 | 2 | 3 |
| 1 | Val/Val | related | 2.15 (0.50) |  |  |  |  |
| 2 | Val/Val | non-related | 3.01 (0.56) | *p* | **< .05** |  |  |
| 3 | Met+ | related | 1.98 (0.46) | *p* | .99 | .55 |  |
| 4 | Met+ | non-related | 4.32 (0.46) | *p* | **< .05** | .34 | **< .001** |

*Note.* *M* = mean; *SE* = standard error of the mean; RT = reaction time; ER = error rate.

*Group differences*

Neither SADNESS scores nor digit span performance differed between Val/Val and Met+ individuals. Since BDNF Val66Met genotype was not associated with digit span performance (Table S9), the intended mediation analysis of EF (as measured by digit span performance) mediating the association between BDNF Val66Met genotype and semantic priming could not be performed.

Table S9.

*Means*, *SDs* and *t*-values for genotype comparisons

|  | BDNF Val66Met | *n* | *M* | *SD* | *t* | *df* | *p* |
| --- | --- | --- | --- | --- | --- | --- | --- |
| SADNESS | Val/Val | 107 | 2.53 | 0.39 | -0.01 | 167 | .83 |
|  | Met+ | 62 | 2.55 | 0.34 |  |  |  |
| digit span(f) | Val/Val | 107 | 5.69 | 1.06 | -0.23 | 167 | .18 |
|  | Met+ | 62 | 5.92 | 1.05 |  |  |  |
| digit span(b) | Val/Val | 107 | 4.16 | 1.07 | -0.28 | 167 | .12 |
|  | Met+ | 62 | 4.44 | 1.15 |  |  |  |

*Note.* f = forward, b = backward.

*Correlational analyses*

SADNESS scores were not significantly associated with any of the examined variables (Table S10). The score of the digit span forward task was only significantly associated with the score of the digit span backward task. In case of the digit span backward task being a stronger representative of EF than the score of the digit span forward task, we only found a significant positive association with the difference in ER priming effects in the masked priming paradigm.

Table S10.

Pearson correlation coefficients between SADNESS, digit span tasks

and priming effects.

|  |  | SADNESS | digit span(f) | digit span(b) |
| --- | --- | --- | --- | --- |
| digit span(f) | *r* | -0.02 |  |  |
| digit span(b) | *r* | 0.01 | 0.38*** |  |
| unmasked RT (N-A) | *r* | 0.04 | -0.06 | -0.07 |
| unmasked RT (N-I) | *r* | 0.10 | -0.05 | 0.01 |
| unmasked ER (N-A) | *r* | 0.00 | 0.07 | 0.04 |
| unmasked ER (N-I) | *r* | -0.01 | 0.02 | 0.06 |
| masked RT (N-A) | *r* | 0.06 | -0.17 | -0.15 |
| masked ER (N-A) | *r* | -0.06 | 0.16 | 0.23* |

*Note.* f = forward, b = backward, RT = reaction times, ER = error rate. All *p*-values were Benjamini-Hochberg corrected for false discovery rate. ****p* < .001; **p* < .05

*Moderation of the association between BDNF Val66Met genotype and performance in digit span tasks by SADNESS scores*

Regression analyses with performance in the digit span forward task as dependent variable and the independent variables BDNF Val66Met genotype and SADNESS did not explain a significant amount of variance (*R^2^* = .01, *F*(2,166) = 0.94, *p* = .39). The additional inclusion of the interaction term did not result in a significant increment in explained variance (*R^2^* = .01, *F*(3,165) = 0.64, *p* = .59).

Regression analyses with performance in the digit span backward task as dependent variable and the independent variables BDNF Val66Met genotype and SADNESS did not explain a significant amount of variance (*R^2^* = .02, *F*(2,166) = 1.24, *p* = .29). The additional inclusion of the interaction term did not result in a significant increment in explained variance (*R^2^* = .02, *F*(3,165) = 1.19, *p* = .31). It is, however, worth mentioning, that the *n* = 14 participants with abnormal behavioral data were individuals that also had a low *mean* digit span backward (*M* = 3.64, *SD* = 1.08) as compared to the group of *N* = 155 individuals we analyzed in the present paper (*M* = 4.32, *SD* = 1.09). Therefore, it is possible that SADNESS has no moderating effect on the association of BDNF Val66Met genotype and digit span backward in individuals that have below average executive functions since these individuals have neither the capacity to perform better nor the possibility to perform much worse than they already do. Thus, inclusion of these individuals could overshadow the small effect of the moderation for individuals showing an average or above average digit span backward.

*Moderation of the association between BDNF Val66Met genotype and differences in masked priming by SADNESS scores*

In the moderation analyses with masked RT priming as dependent variable, BDNF Val66Met genotype and SADNESS together with the covariate age did not explain a significant amount of variance (*R^2^* = .03, *F*(3,165) = 1.94, *p* = .13). Further, the introduction of the interaction term yielded no significant increase in explained variance (*R^2^* = .05, *F*(4,164) = 2.10, *p* = .08).

In the regression analyses with masked ER priming as dependent variable, the independent variables BDNF Val66Met genotype and SADNESS explained a significant amount of variance (*R^2^* = .04, *F*(2,166) = 3.83, *p* < .05). However, the additional inclusion of the interaction term did not result in a significant increment in explained variance (*R^2^* = .05, *F*(3,165) = 2.68, *p* < .05, *b* = -1.55, *t*(165) = -0.64, *p* = 0.52).

Exclusion of the individual with a mean ER of 48.13 % across all trials of the masked priming paradigm, however, yielded different results. This participants presumably guessed in the lexical decision task of the masked priming paradigm, which is why one cannot assume an adequate assessment of priming effects.

Regression analyses with masked RT priming as dependent variable, BDNF Val66Met genotype and SADNESS together with the covariate age did also not explain a significant amount of variance (*R^2^* = .04, *F*(3,164) = 2.24, *p* = .09). However, the introduction of the interaction term yielded a significant increase in explained variance (*R^2^* = .06, *F*(4,163) = 2.70, *p* < .05, *b* = 20.37, *t*(163) = 1.98, *p* < .05). Low SADNESS scores were associated with larger priming effects in Val/Val homozygotes while Met+ carriers showed almost no priming in case of low SADNESS scores. Differences between Val/Val homozygotes and Met+ individuals declined with increasing SADNESS scores. Additionally, SADNESS showed a stronger influence in Met+ individuals as compared to Val/Val homozygotes (Fig S1).

**Fig. S1** SADNESS significantly moderated the association between BDNF Val66Met genotype and masked RT priming effects. High/low refers to mean +/- 1 SD. Estimates are based on setting the covariate to its sample mean. Masked RT priming corresponds to differences in reaction times (in ms) between trials with semantically related and trials with semantically non-related prime-target-pairings

In the regression analyses with masked ER priming as dependent variable, the independent variables BDNF Val66Met genotype and SADNESS explained a significant amount of variance (*R^2^* = .05, *F*(2,165) = 3.91, *p* < .05). However, the additional inclusion of the interaction term did not result in a significant increment in explained variance (*R^2^* = .05, *F*(3,164) = 2.78, *p* < .05, *b* = -1.16, *t*(164) = -0.74, *p* = 0.46).
